# New Therapeutic Candidates for the Treatment of *Malassezia pachydermatis -*Associated Infections

**DOI:** 10.1038/s41598-020-61729-1

**Published:** 2020-03-17

**Authors:** Angie Sastoque, Sergio Triana, Kevin Ehemann, Lina Suarez, Silvia Restrepo, Han Wösten, Hans de Cock, Miguel Fernández-Niño, Andrés Fernando González Barrios, Adriana Marcela Celis Ramírez

**Affiliations:** 1 Instituto de Biotecnología (IBUN), Facultad de Ciencias, Universidad Nacional de Colombia, Bogotá, 11001 Colombia; 2 Grupo de Investigación Celular y Molecular de Microorganismos Patógenos (CeMoP), Departamento de Ciencias Biológicas, Universidad de los Andes, Bogotá, 111711 Colombia; 3 Grupo de Diseño de Productos y Procesos (GDPP), Departamento de Ingeniería Química, Universidad de los Andes, Bogotá, 111711 Colombia; 4 Structural and Computational Biology Unit, European Molecular Biology Laboratory (EMBL), Heidelberg, 69117 Germany; 5 Collaboration for joint PhD degree between EMBL and Heidelberg University, Faculty of Biosciences, Heidelberg, Germany; 6 Laboratorio de Micología y Fitopatología (LAMFU), Departamento de Ingeniería Química, Universidad de los Andes, Bogotá, 111711 Colombia; 7 Microbiology, Department of Biology, Utrecht University, Utrecht, The Netherlands

**Keywords:** Fungal infection, Antifungal agents

## Abstract

The opportunistic pathogen *Malassezia pachydermatis* causes bloodstream infections in preterm infants or individuals with immunodeficiency disorders and has been associated with a broad spectrum of diseases in animals such as seborrheic dermatitis, external otitis and fungemia. The current approaches to treat these infections are failing as a consequence of their adverse effects, changes in susceptibility and antifungal resistance. Thus, the identification of novel therapeutic targets against *M. pachydermatis* infections are highly relevant. Here, Gene Essentiality Analysis and Flux Variability Analysis was applied to a previously reported *M. pachydermatis* metabolic network to identify enzymes that, when absent, negatively affect biomass production. Three novel therapeutic targets (i.e., homoserine dehydrogenase (MpHSD), homocitrate synthase (MpHCS) and saccharopine dehydrogenase (MpSDH)) were identified that are absent in humans. Notably, L-lysine was shown to be an inhibitor of the enzymatic activity of MpHCS and MpSDH at concentrations of 1 mM and 75 mM, respectively, while L-threonine (1 mM) inhibited MpHSD. Interestingly, L- lysine was also shown to inhibit *M. pachydermatis* growth during *in vitro* assays with reference strains and canine isolates, while it had a negligible cytotoxic activity on HEKa cells. Together, our findings form the bases for the development of novel treatments against *M. pachydermatis* infections.

## Introduction

The yeast *M. pachydermatis* is part of the skin microbiota of domestic and wild animals and behaves as an opportunistic pathogen causing external otitis and seborrheic dermatitis in dogs and cats. Particular conditions such as the presence of lipid-rich microenvironments, a local imbalance of the natural microbiota and altered immune states favor these infections^1^. Dermatologic infections caused by *M. pachydermatis* often exhibit a chronic (recurrent) course and their treatment can be complicated due to the ability of this yeast to form biofilms^1^. In addition, *M. pachydermatis* causes bloodstream infections in preterm infants or in individuals with immunodeficiency disorders. These infections are related to contamination of medical devices such as catheters, the transmission through medical staff and the administration of lipids through intravenous way^2,3^. Recently, several factors contributing to *M. pachydermatis* virulence have been determined, which include the production of proteinases, phospholipases, hyaluronidases, and chondroitin-sulfatases^4^.

Currently, five classes of antifungal agents are used orally, topically or intravenously for the treatment of fungal infections. The first class is formed by the azoles (ketoconazole, itraconazole, clotrimazole, miconazole, and voriconazole) that interfere with ergosterol synthesis by interacting with sterol-14α-demethylase. The second and third class, i.e. allylamines (terbinafine and naftifine) and polyenes (nystatin, natamycin, and amphotericin B) also target ergosterol by interfering with its synthesis by inhibiting squalene sterol-14α-demethylase and by producing pores in membranes by binding ergosterol, respectively. Echinocandins (caspofungin, micafungin, and anidulafungin) are the only available antifungal drugs targeting the cell wall, acting as noncompetitive inhibitors of the β-(1,3)-D-glucan synthase enzyme complex. The fifth class of anti-fungals are formed by the pyrimidine analogs like flucytosine that interfere with pyrimidine metabolism and RNA/DNA and protein synthesis^2,3,5–8^. Azoles and amphotericin B are mainly used to treat *M. pachydermatis* infections^6,9^. These infections have been classified as chronic, which may require prolonged treatment and thereby causing adverse effects^1,3,6,8,10^. The increase in incidence of azole-resistant strains and the number of therapeutic failures in animals^2,11^ also underline the importance to identify new therapeutic targets for the treatment of *M. pachydermatis* infections.

Searching therapeutic targets through metabolic network reconstructions has been proposed as a strategy to control the virulence of pathogens^12,13^. A frequently used approach is Gene Essentiality Analysis (GEA) that analysis the impact of *in silico* deletions to identify potentially essential genes for growth of an organism^12,14^. This approach provides useful information about the metabolism of target organisms, which can be used to nominate therapeutic candidates^13,15,16^. The aim of this study was to identify novel therapeutic targets for *M. pachydermatis* by GEA and to confirm their potential by assessing the inhibitory capacity of inhibitors. Results indicate that MpHSD, MpHCS, MpSDH are targets to treat *M. pachydermatis* infections.

## Results

### Novel potential therapeutic targets against *M. pachydermatis*

Curation of the metabolic network of *M. pachydermatis*^17^ identified 19 metabolites that cannot be produced and/or consumed by any of the reactions or imported/exported through any of the available uptake/secretion pathways in the model (Table S1). This was solved by adding 21 reactions (Table S2) and adjusting the upper and lower bounds of another 18 reactions (Table S3). The Flux Balance Analysis (FBA) of the curated network had a biomass flux of 3.13 and consisted of 45% active fluxes (reactions with a flux >0.01 mmol gDW^−1^h^−1^). Flux Variability Analysis (FVA) was performed to identify the flux range variability of each reaction. FVA revealed that 42.3% of the reactions of the *M. pachydermatis* metabolic network showed a difference between the maximum and minimum fluxes other than zero. These reactions represent the defined space of flux distributions of the optimal solution. This means that these reactions do not affect the overall flux of biomass as alternative pathways could be used to fulfill the objective function. This natural flexibility has been associated to the ability of organisms to face environmental changes (i.e. fitness of the cell)^18^. In contrast, reactions with a low range of plasticity (that is, reactions with a difference value between maximum and minimum fluxes equal to zero) or essential reactions related to the biomass reaction were around 16.4%. These essential reactions are catalyzed by 606 enzymes in the network.

GEA was applied to identify potential therapeutic targets for *M. pachydermatis*. *In silico* deletion of candidate genes should have a negative effect on the growth of the organism according to FBA performed in this study. The 606 enzymes catalyzing essential reactions in the network were grouped into 602 enzyme clusters by sequence similarity. A total of 67 enzyme clusters were identified as potential targets for growth inhibition based on the fact that their *in-silico* deletion resulted in a reduction of the flow of biomass ≥70%. This same procedure was also performed in *M. furfur* (data not shown) showing an overlap of 15 enzymes that are predicted to inhibit growth. Only three of these 15 enzymes (i.e. Imidazoleglycerol-phosphate dehydratase (IGPD), 6,7-dimethyl-8-ribitilumazine synthetase (RIBH) and riboflavin synthetase (RIB)) did not have a significant match with a human protein (Table 1). However, these enzymes were not selected for further studies as no commercial kits for their quantification are available. The *M. pachydermatis* enzymes homoserine dehydrogenase (MpHSD), homocitrate synthase (MpHCS) and saccharopine dehydrogenase (MpSDH) were selected for further studies as they showed a similarity <20% with a human protein (Table 1), there are no enzyme homologues reported in mammals and there are inhibitors of orthologues already reported in the literature for instance in *Schizosaccharomyces pombe* (HCS)^19^, *Corynebacterium glutamicum* (HSD)^20^, and *S. cerevisiae* (SDH)^21^.In fact, a literature review identified 58 inhibitors for HSD, 62 for HCS and 67 for SHD.

**Table 1:** Potential therapeutic targets against *M. pachydermatis*.

EC Number	Abbreviature	Enzyme	Human Match	% Protein similarity
4.2.1.19	IGPD	Imidazoleglycerol-phosphate dehydratase	NA	NA
2.5.1.78	RIBH	6,7-Dimethyl-8-ribityllumazine synthase	NA	NA
2.5.1.9	RIB	Riboflavin synthase	NA	NA
**2.3.3.14**	**HCS**	**Homocitrate synthase**	**HMGCL**	**14.4**
**1.1.1.3**	**HSD**	**homoserine dehydrogenase**	**PSB5**	**18.31**
**1.5.1.7; 1.5.1.10**	**SDH**	**Saccharopine dehydrogenase**	**F8WE53**	**19.71**
4.2.1.36	HACN	Homoaconitate hydratase	ACON	21.36
1.1.1.41; 1.1.1.42	IDH	Isocitrate dehydrogenase	IDH3A	32.72
1.14.13.70	CYP51A1	Lanosterol 14 alpha-demethylase	CP51A	36.48
2.7.4.22	UK	Uridylate kinase	KCY	40.7
1.3.1.-	ERG4/ERG24	Ergosterol biosynthesis sterol reductase ERG4/ERG24	LBR	41.18
5.4.2.8	PMM	Phosphomannomutase	H3BV55	51.06
6.4.1.1	PC	Pyruvate carboxylase	PC	51.43
3.6.1.9	ITPA	Inosine triphosphate pyrophosphatase	ITPA	52.91
6.3.4.5	AS	Argininosuccinate synthase	ASSY	55.3

The *in-silico* deletion of these enzymes resulted in a decrease in the production of biomass of at least 30%. The percentage of similarity and human match to the human proteome is also shown. Prioritized enzymes are shown in bold and underline.

### L-lysine and L-threonine are potential inhibitors of MpHCS, MpHSD and MpSDH

The genes encoding MpHSD, MpHCS and MpSDH were heterologously expressed to assess their enzymatic activity in the presence of potential inhibitors. To this end, HIS-tag fusion genes were cloned into the expression vectors pET6xHN-N or pET6xHN-C (Supplementary Fig. S1) and introduced into *E. coli BL21*. SDS-PAGE revealed bands of 81, 51, and 43 kDa for MpHSD, MpHCS enzyme and MpSDH, respectively (Supplementary Figs. S1, S2). The additional bands likely correspond to proteins with histidine residues of *E. coli* BL21 that were co-purified with the histidine-tagged recombinant protein^22,23^.

**Figure 1:**
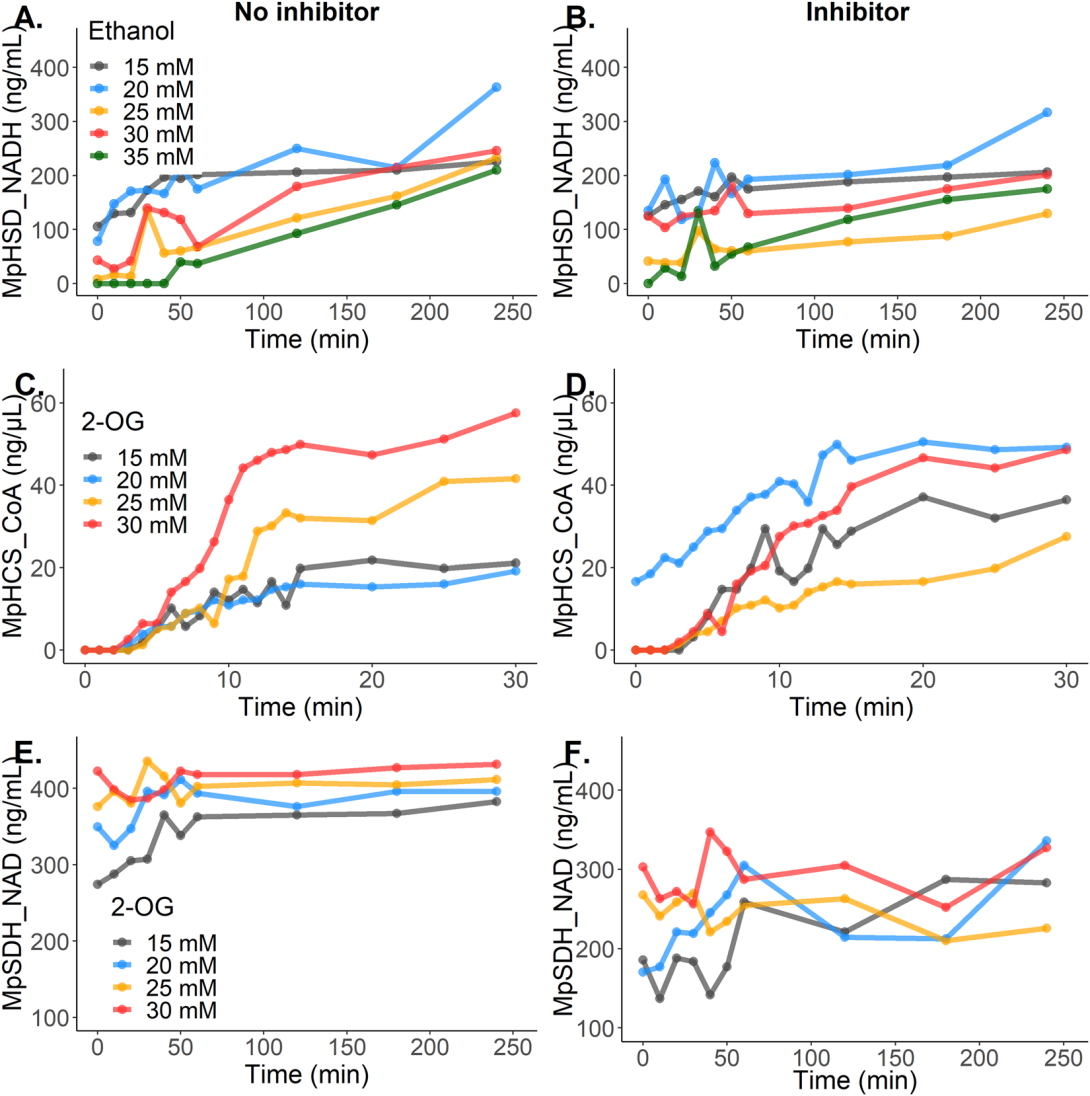
Evaluation of the inhibitory capacity of amino acids upon candidates as therapeutic targets. (**A**) Enzymatic activity of HSD with ethanol as variable substrate (colored lines), NADH as reaction indicator and detected for four hours. (**B**) Enzymatic activity of HSD adding L-threonine 1 mM as an inhibitor. (**C**) Enzymatic activity of HCS with 2-OG as variable substrate (colored lines), CoA as reaction indicator and detected for thirty minutes. (**D**) Enzymatic activity of HCS adding L-lysine 1 mM as an inhibitor. (**E**) Enzymatic activity of SDH with 2-OG as variable substrate (colored lines), NAD as reaction indicator and detected for four hours. (**F**) Enzymatic activity of SDH adding L-lysine 75 mM as inhibitor. Representative results of two biological replicates.

MpHSD, MpHCS and MpSDH activity was monitored in inhibition assays using NAD/NADH^24^ and coenzyme A^25^ quantification (see Material and Methods). The three enzymes carried out the expected catalytic activity in the absence of inhibitors (Fig. 1A,C,E; Supplementary Fig. S3A,C,E), while inhibitors reduced enzymatic activity (Fig. 1B,D,F; Supplementary Fig. 3B,D,F). The highest final concentrations of formed NADH were 363.3 ng/mL (Fig. 1A) and 316.42 ng/mL (Fig. 1B), respectively, in the first MpHSD activity test without and with 1 mM L-threonine as inhibitor. In the case of MpHCS activity, highest values of CoA were 57.56 ng/µL (Fig. 1C) in absence and 49.25 ng/µL in presence of the 1 mM L-lysine as inhibitor (Fig. 1D). Highest final concentrations of formed NAD were 431.2 ng/mL (Fig. 1E) without inhibitor and 336.1 ng/mL (Fig. 1F) with 75 mM L-lysine as inhibitor in the case of MpSDH. The second assays or biological replicates showed the same concentration differences in the absence and presence of inhibitor for each enzyme (Supplementary Fig. S3A–F), showing that threonine is an inhibitor of MpHSD and lysine of MpHCS and MpSDH. It should be noted that in the first trial MpHCS activity was higher in the presence than in the absence of inhibitor when 15 and 20 mM 2-oxoglutaric acid (2-OG) was used. This may have been caused by a higher actual 2-OG concentration or by a L-lysine concentration that was too low to inhibit (Fig. 1C,D). Note that these values were included in the Lineweaver-Burk diagrams (Supplementary Fig. S4C) still enabling the kinetic parameters to be determined (Table 2).

**Table 2:** Kinetic parameters for enzymatic activity of MpHSD, MpHCS and MpSDH in the presence and absence of the inhibitors L-threonine or L-lysine.

Enzyme	Condition	K_m_ (mM)	V_max_ (mmol/min) for condition	V_max_ for both assays (mmol/min)
MpHSD	Without inhibitor	0.59 ± 2.1e-02	1.56e-06 ± 5.4e-08 (mM/min)	1.98e-06 ± 5.9e-07 (mM/min)
	With inhibitor	1.41 ± 6.2e-02	2.40e-06 ± 1.2e-06 (mM/min)	
MpHCS	Without inhibitor	0.35 ± 2.1e-01	1.24e-06 ± 1.7e-06	2.11e-06 ± 2.9e-06
	With inhibitor	1.36 ± 3.7e-02	2.98e-06 ± 2.1e-06	
MpSDH	Without inhibitor	0.25 ± 7.1e-02	7.50e-09 ± 3.5e-09	7.80e-09 ± 4.0e-09
	With inhibitor	0.83 ± 2.4e-01	8.06e-09 ± 4.3e-09	

Data are shown with standard deviations.

Results of two biological replicates.

Kinetic parameters based on the *Michaelis-Menten* model^26,27^ and *Lineweaver-Burk* diagram^28^ showed competitive inhibition of MpHSD by 1 mM L-threonine, while in the case of MpHCS and MpSDH 1 mM and 75 mM L-lysine, respectively, showed competitive inhibition (Table 2). The K_m_ of MpHSD with and without inhibitor was 1.41 ± 6.2e-02 and 0.59 ± 2.1e-02 mM, respectively, while V_max_ was not affected with a value of 1.98e-06 ± 5.9e-07 (mM/min) (Table 2, Supplementary Fig. S4A,B). K_m_ of MpHCS was 0.35 ± 2.1e-01 and 1.36 ± 3.7e-02 in absence and presence of inhibitor, respectively, while V_max_ was not affected with a value of 2.11e-06 ± 2.9e-06 (Table 2, Supplementary Fig. S4B,C). Lastly, MpSDH had an increase in the K_m_ of 0.25 ± 7.1e-02 without inhibitor to 0.83 ± 2.4e-01 with inhibitor, whereas also in this case V_max_ was not affected by the inhibitor showing a value of 7.80e-09 ± 4.0e-09 (Table 3, Supplementary Fig. S4C,D). Together, these results indicate that L-threonine and L-lysine inhibit the enzymatic activity in a competitive way because these interfere with correct substrate-enzyme complex formation which is reflected in the increase of the K_m_ value.

**Table 3:** MICs (mg/mL) for *Malassezia* spp. evaluated with L-lysine and L-threonine and values for AB and Flz control (µg/mL).

Strains		MIC L-lysine		MIC L-threonine	MIC to AB^a^
**Microdilution assays**					
*M. pachydermatis* CBS 1879^b^		3.1		>25	0.25
*M. pachydermatis* C1^c^		3.1		>25	—
*M. pachydermatis* M1^c^		3.1		>25	—
*M. pachydermatis* N1^c^		3.1		>25	—
Atypical *M. furfur*^b^		3.1		>25	—
*M. sympodialis* CBS 7222^b^		3.1		>25	—
*M. furfur* CBS 1878^b^		1		>25	4
*C. krusei* ATCC 6258^b^		—		—	2
*C. parapsilosis* ATCC 22019^b^		—		—	0.25
**E-test® method**					
**Strain**	**Flz**^**a**^		**AB**^**a**^		
*M. pachydermatis* CBS 1879^b^	48–64		1–2		
*M. pachydermatis* N1^c^	64–128		3–6		
*M. pachydermatis* M1^c^	8–16		3–4		
*M. pachydermatis* C1^c^	4		8		
*M. furfur* CBS 1878^b^	3–4		>32		
*M. sympodialis* CBS 7222^b^	0.75–1.5		2		

^a^Reference values (µg/mL) amphotericin B (AB) 0.125–8 and fluconazole (Flz) ≤64.

^b^Reference strain.

^c^Canine isolate.

(—) No tested.

>No inhibition, likely MIC higher.

Microdilution assays were performed in triplicate.

E-test^®^ was done for duplicate and the results are showed among ranks.

### L-lysine and L-threonine inhibit growth of *M. pachydermatis*

**Figure 2:**
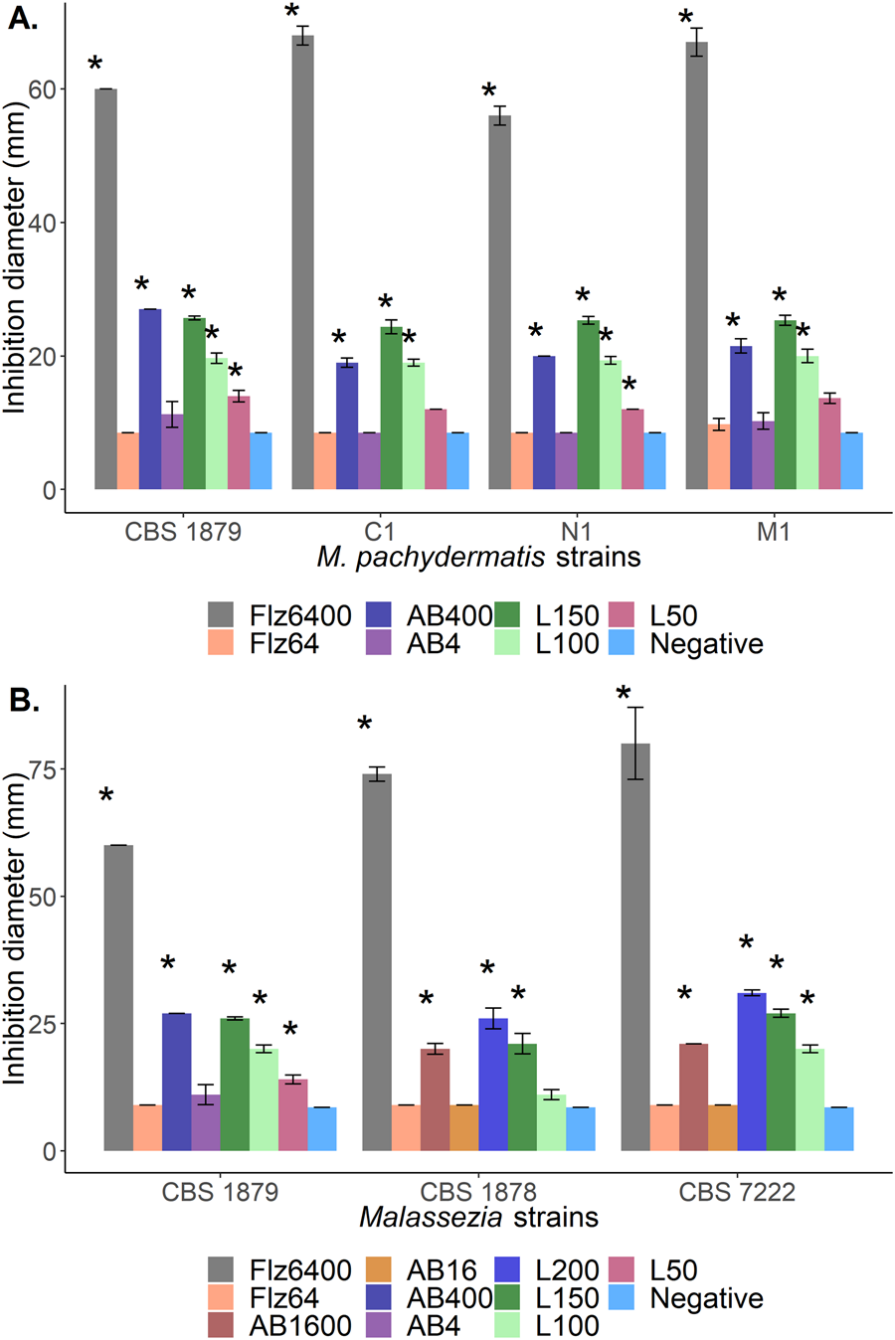
Agar diffusion assays to evaluate the inhibitory capacity of L-lysine upon the growth of *M. pachydermatis* and *Malassezia* strains. (**A**) The diameter of the inhibition zones of three different concentrations of L-lysine upon *M. pachydermatis* isolates. (**B**) Diameter of the inhibition zones measured for three different concentrations of L-lysine for *M. pachydermatis*, *M. furfur* and *M. sympodialis*, respectively. experiments were performed in triplicate and the results are shown with standard deviation. *Differences between the diameter of the zone of inhibition significantly different (p-value < 0.05) from the negative control. AB, amphotericin B; Flz, fluconazole; L, L-lysine. Concentrations in mg/mL for Lysine and µg/mL for AB and FLZ.

Microdilution and agar well diffusion assays were used to determine the effect of L-lysine and L-threonine on growth of reference strains of *Malassezia* spp. and canine isolates of *M. pachydermatis* (Table 4). The MIC of L-lysine was the same (i.e. 3.1 mg/mL) for all reference strains and canine isolates of *M. pachydermatis*, but not for the atypical *M. furfur* strain that was shown to be more susceptible with a MIC of 1 mg/mL of L-Lysine (Table 3). In contrast, L-threonine did not show inhibition for any strain at 25 mg/mL, which was the maximum concentration that could be evaluated due to the water solubility limit of the reagent used (Table 3). Agar diffusion assays did not show inhibited growth of any of the strains at levels of threonine up to 50 mg/mL (results not shown). In contrast, growth of *M. pachydermatis* strains was inhibited at ≥50 mg/mL lysine (Fig. 2A), while growth inhibition of *M. sympodialis* and *M. furfur* was observed above 100 mg/mL (Fig. 2A,B). More specifically, *M. pachydermatis* isolates CBS 1879 and M1 showed significant inhibition at 50 mg/mL L-lysine, whilst isolates C1, N1 were inhibited at 100 mg/mL. Growth of *M. sympodialis* strain CBS 7222 and the *M. furfur* strain CBS 1878 were only significantly inhibited at ≥150 mg/mL L-lysine (Fig. 2A). The atypical *M. furfur* strain was not evaluated by agar diffusion as grew poorly on culture media. As comparison, growth inhibition was evaluated with two antifungals. The E-test^®^ indicated that *M. pachydermatis* strain CBS 1879 strain was most sensitive for amphotericin B (MIC 1–2 µg/mL) and the C1 strain for fluconazole (MIC 4 µg/mL) (Table 3). No inhibition of growth was observed at low concentrations of amphotericin B (4 or 16 µg/mL) and fluconazole (64 µg/mL), while growth was inhibited in all cases with higher concentrations used (400 or 1600 µg/mL) (Fig. 2). Together, results show that L-lysine inhibits growth of *Malassezia* spp.

**Table 4:** Strains and plasmids used in this study.

Strain	Description	Reference
*Malassezia pachydermatis* CBS 1879	Reference strain. Genome Sequenced: NCBI: txid 77020.	^101,102^
*Malassezia furfur* CBS 1878	Reference strain	^101^
Atypical *Malassezia furfur* 4DS	Reference strain	^103^
*Malassezia sympodialis* CBS 7222	Reference strain	^101^
*Candida krusei* ATCC 6258	Reference strain	^104^
*Candida parapsilosis* ATCC 22019	Reference strain	^104^
*Malassezia pachydermatis* C1	Canine isolate from the collection of the Cellular and Molecular research group of Microorganisms Pathogens (CeMoP, acronym in Spanish). From ears of a 2-years old female cocker spaniel dog.	^105^
*Malassezia pachydermatis* N1	Canine isolation from the collection of the CeMoP. From ears of a 9-years old female cocker spaniel dog.	^105^
*Malassezia pachydermatis* M1	Canine isolation from the collection of the CeMoP. From the ears of a male 1-year old Shih-Tzu dog.	^105^
DH5α-pUC57-*mpsdh*	*Escherichia coli* DH5α strain transformed with pUC57-*mpsdh*	This study
DH5α-pET6xHN-C-*mpsdh*	*Escherichia coli* DH5α strain transformed with pET6xHN-C-*mpsdh*	This study
BL21-pET6xHN-C-*mpsdh*	Strain *Escherichia coli* BL21(DE3) transformed with pET6xHN-C-*mpsdh*	This study
BL21-pET6xHN-N-*mphcs*	Strain *Escherichia coli* BL21(DE3) transformed with pET6xHN-N-*mphcs*	This study
BL21-pET6xHN-N-*mphsd*	Strain *Escherichia coli* BL21(DE3) transformed with pET6xHN-N-*mphsd*	This study
pET6xHN-C	Plasmid containing an IPTG inducible promoter system (T7/lac promoter) for high-level expression, a C-terminal 6xHN tag and conferring ampicillin resistance	^106^
pET6xHN-N	Plasmid containing an IPTG inducible promoter system (T7/lac promoter) for high-level expression, an N-terminal 6xHN tag and conferring ampicillin resistance	^106^
pUC57-*mpsdh*	Plasmid pUC57 containing a synthetic expression cassette encoding the enzyme saccharopine dehydrogenase (MpSDH) of *M. pachydermatis* flanked by *Nco*I and *Not*I restriction sites and conferring ampicillin resistance.	This study
pET6xHN-C-*mpsdh*	Plasmid pET6xHN-C containing a synthetic expression cassette encoding the enzyme saccharopine dehydrogenase (MpSDH) of *M. pachydermatis* flanked by *Nco*I and *Not*I restriction sites, conferring ampicillin resistance and with C-terminal 6xHN tag.	This study
pET6xHN-N-*mphcs*	Plasmid pET6xHN-N containing an amplified expression cassette encoding the enzyme homocitrate synthase (MpHCS) of *M. pachydermatis* flanked by HCSF and HCSR primers sites, conferring ampicillin resistance and with C-terminal 6xHN tag.	This study
pET6xHN-N-*mphsd*	Plasmid pET6xHN-N containing an amplified expression cassette encoding the enzyme homoserine dehydrogenase (MpHSD) of *M. pachydermatis* flanked by HSDF and HSDR primer sites, conferring ampicillin resistance and with C-terminal 6xHN tag.	This study

### The amino acid L-lysine has a low cytotoxic effect on HEKa cells

A MTT viability assay was performed using HEKa^29^ (Primary Epidermal Keratinocytes) cells to determine if L-lysine is cytotoxic to human cells. L-lysine was shown to have a mild cytotoxic activity. Cell viability was 100% in the absence of lysine while 90.5% ± 0.06%, 79.3% ± 0.3 and 67.74% ± 0.08% of the cells survived in the presence of 100, 150, and 200 mg/mL L-lysine, respectively (Supplementary Fig. S5).

## Discussion

In this study, a metabolic network of *M. pachydermatis* CBS 1879^17^ was curated to identify potential novel therapeutic targets against *M. pachydermatis* infections. The improved network shows fluxes that are in agreement with those in other yeasts such as *S. cerevisiae*^30^ and *Ustilago maydis*^31^. FVA and GEA revealed several genes including MpHSD, MpHCS, and MpSDH whose *in-silico* deletions were predicted to result in a reduction in biomass formation.

HSD is involved in the aspartate route resulting in the L-amino acids lysine, threonine, cysteine and methionine. This enzyme catalyzes the third reversible reaction in this pathway producing L-homoserine^20,32^. Notably, this “sulfur assimilation pathway” is present in the fungal kingdom, but not in humans^33,34^. HCS and SDH catalyze the first and last step of the α-aminoadipate pathway that results in L-lysine biosynthesis. The former catalyzes the conversion of 2-OG into homocitrate^35,36^, while the latter catalyzes the reversible conversion of saccharopine to L-lysine and α-ketoglutarate (α-Kg) using NAD as an oxidant^37^. These two enzymes are also absent in mammals. In the latter, the bifunctional enzyme alpha-aminoadipic semialdehide synthase (EC 1.5.2.8) is present that has a saccharopine dehydrogenase domain, but it is not inhibited by L-lysine^38^. In contrast, HCS and SDH are regulated by feedback inhibition by an excessive amount of L-lysine, while HSD is similarly regulated by the end products of the aspartate route including L-threonine^32,39,40^.

The K_m_ of MpHSD of 0.59 mM was similar to that of the HSDs of *E. coli* (0.68 mM), *Staphylococcus aureus* (0.69 mM) and *Sulfolobus tokodaii* (0.54 mM)^32,41,42^. Moreover, the kinetic parameters indicated that L-threonine is a competitive inhibitor for MpHSD activity (1.41 mM), as was shown in *Brevibacterium flavum* (at 3.3 mM)^43^, *E. coli* (at 0.08 mM or 0.5 mM)^44,45^ and *Corynebacterium glutamicum* (at 0.5 mM or 2 mM)^20,46^. The K_m_ of HCS ranges from 0.044 to 1.3 mM for organisms like *Thermus thermophiles, Candida albicans, Schizosaccharomyces pombe and S. cerevisiae*^19,35,39,47–50^. Thus, the affinity constant of MpHCS of 0.35 mM is within this reported range. L-lysine was shown to compete with the substrate 2-OG increasing the K_m_ to 1.36 mM. The inhibitory effect of L-lysine has been shown to be due to binding to free enzyme in *S. cerevisiae*, while the competitive inhibition of lysine versus α-KG (at a low concentration) can be explained by active-site and not allosteric inhibition^51^. The K_m_ of SDH ranges between 0.11 and 0.66 mM in *S. cerevisiae, Pichia guilliermondii* and *Candida maltosa*^21,40,52–55^, while that of MpSDH again is in this range with 0.25 mM. The reductive condensation of α-ketoglutarate and lysine with SDH can be inhibited by concentrations of lysine >60 mM in *S. cerevisiae*^21,56^. In this study, the *Michaelis-Menten* constant changed but not the V_max_ indicating that L-lysine at 75 mM was a competitive inhibitor for 2-OG.

We assessed whether the MpHSD, MpHCS, MpSDH inhibitors threonine and lysine can inhibit growth of the reference strains *M. pachydermatis* CBS 1879*, M. furfur* CBS 1878, *M. sympodialis* CBS 7222 and atypical *M. furfur*, and three *M. pachydermatis* canine isolates. L-lysine indeed reduced the growth of these strains most likely due to feedback inhibition and specific repression of the synthesizing enzymes. Similar observations have been reported in studies with *S. cerevisiae*^57–59^ and *P. chrysogenum*^60^, where increases on the concentration of lysine in the medium resulted in cell death. This was observed when lysine was used as the sole source of nitrogen and consequently it is not properly metabolized. This toxicity may also be due to negative regulation of SDH and HCS that can generate accumulation or absence of intermediate metabolites^61^. Moreover, growth repression may be due to phenomena like general control, which involves a simultaneous derepression of one or more enzymes of several unrelated amino acid biosynthetic pathways, in response to external imbalance^61,62^, and it can contribute to the internal imbalance of the amino acid pools even further, causing cell death^63^. Thus, the inhibition of *M. pachydermatis* growth, as well as other *Malassezia* strains by L-lysine could be by, at least, two mechanisms of regulation of the α-aminoadipate pathway.

It was not possible to determine the inhibitory capacity of L-threonine on growth of the *Malassezia* strains due to the limitations of water solubility^64^. Previous studies showed that feedback inhibition of HSD by 5 mm L-threonine reduced growth of *Pseudomonas putida* by 22%^65^. In contrast, no growth inhibition was observed in the case of *Acetobacter aceti* at 10 mM L-threonine despite inhibition of HSD^66^. There were differences in the concentrations of L-lysine, amphotericin B (AB), and fluconazole (Flz) that inhibited growth of the *Malassezia* strains between the microdilution and agar well diffusion assays. This might be due to differences in lipidic composition of the broth and agar cultivation mediums, which influences the antifungal sensitivity^67,68^, despite of this, the agar well diffusion method has been shown suitable to evaluate the inhibition of natural extracts on *M. furfur*^69^ and for both tests, L-lysine presented growth inhibition.

The MTT cell viability assay showed that L-lysine concentrations up to 150 mg/mL is not cytotoxic to Heka cells considering the definition in ISO 10993-5^70^ that states that any solution or item that reduces the cell viability to 70% or less has a cytotoxic potential^70^. Nonetheless, a L-lysine concentration of 200 mg/mL did reduce the cell viability to 67.74%, exhibiting a cytotoxic potential activity. The cytotoxic activity of the most concentrated L-lysine solution could be explained by the cationic properties of L-lysine as has been reported^71,72^. Morgan *et al*., (1989) suggested that the cytotoxicity displayed by cationic macromolecules is strongly related to the high density of multivalent interactions or bindings with anionic groups at the surface of the cell^72^. Although these studies were performed with cationic polyaminoacids, it could be deduced that a high concentration of cationic amino acids, as L-lysine, could interact with the cell surface as well, exhibiting a similar cytotoxicity as shown in Choksakulnimitr’s research^71^. Overall, our results suggest that L-lysine could be used as a potential treatment against *M. pachydermatis* associated-diseases. It is important to mention that the concentrations required for growth inhibition could limit their clinical utility. Thus, L-lysine could be used to treat dermatological infections instead of sepsis where higher concentrations will be required. Additional cytotoxicity, pharmacokinetic and toxicodynamic studies will be needed to confirm its potential as a novel pharmaceutical product and possible treatment against *M. pachydermatis* infections.

## Conclusions

In this study, *in silico* modeling together with *in vitro* analysis identified and nominated three essential genes as novel therapeutic targets against *M. pachydermatis*-associated infections. Their activity can be inhibited *in vitro* using L-threonine or lysine. Interestingly, L-lysine was shown to be able to reduce also the growth of *Malassezia* spp., and presented no cytotoxic activity in keratinocytes at a concentration ≤150 mg/mL. Further studies will be required to evaluate clinical application including analysis of pharmacokinetic/ADME-Tox (Absorption, Distribution, Metabolism, and Excretion – Toxicology), pharmacodynamics, and biopharmacy studies.

## Materials and Methods

### Strains, plasmids, and media composition

Strains and plasmids used in this study are listed in Table 4. Cells of *E. coli* were grown and maintained in Luria Bertani (LB) agar or broth containing 10 g/L Tryptone [Oxoid], 5 g/L yeast extract [Oxoid], 10 g/L NaCl [Merk], 14.8 g/L agar bacteriological [Scharlau] and 1 mL/L ampicillin [Sigma-Aldrich]. Growth of *Malassezia* yeasts as well as agar diffusion assays were carried out in modified Dixon agar medium (mDixon) containing 36 g/L Mycosel [DB], 36 g/L yeast extract [Oxoid], 20 g/L Oxgall [DB], 2 mL/L glycerol [Sigma-Aldrich], 2 mL/L oleic acid [Carlo Erbba], 10 mL/L Tween 40 [Sigma-Aldrich], and 0.5 g/L chloramphenicol [Colmed International, Sigma]. Microdilution tests were performed in modified Sabouraud broth (mSabouraud) containing 30 g/L Sabouraud broth [Scharlau], 5 ml/L Tween 40 [Sigma-Aldrich], 5 mL/L Tween 60 [Sigma-Aldrich] and 0.25 g/L chloramphenicol [Colmed International, Sigma]. *Candida* spp. were grown in 30 g/L Sabouraud agar [Scharlau]) containing 0.25 g/L chloramphenicol [Colmed International, Sigma].

### Metabolic network curation

The metabolic network of *M. pachydermatis*^10^ was manually curated using the biomass objective function as a starting point. First, metabolites with no production, accumulation and absorption problems were identified using standard protocols (network gap filling)^15^. Subsequently, a review and search of reactions in specialized literature and in databases such as KEGG^73^ and BioCyc^74^ was carried out, allowing the identification of missing reactions in the metabolic pathways. Those reactions were added in the network if the enzyme that catalyzed each reaction was present in the *M. pachydermatis* genome. Furthermore, FBA was run using GAMS software^75^ and CPLEX as the optimization solver^76^.

### Gene essentiality analysis

The curated metabolic network of *M. pachydermatis* was used to perform the Genetic Essentiality Analysis (GEA). Initially, the flux of biomass was defined as objective function as we wanted to identify essential genes related to growth^10^. Then, the network was analyzed using Flux Balance Analysis (FBA) and Flux Variability Analysis (FVA). FBA was used to identify the core reactions, enzymes, and genes related to biomass production, while FVA was used to determine the plasticity^77^. Here, FVA was used to determine the feasible region of the FBA problem, which is the variability range and the plasticity of each flux to satisfy a fixed biomass value^78^. After that, the flux of each reaction was minimized and maximized, as follows:

1 $$Maximize\,\,\& \,{minimize}\,z={v}_{j}$$

*Subject to*

2 $${S}_{i,j}\ast {v}_{j}=0$$

3 $$0\le {v}_{j}\le 1000\frac{mmol}{gDW\ast h}\,if\,j\,is\,irreversible$$

4 $$-1000\le {v}_{j}\le 1000\frac{mmol}{gDW\ast h}\,\,if\,j\,is\,reversible$$

5 $${v}_{Biomass}={v}_{Biomass}^{FBA}$$

where z is the variable to optimize, ${v}_{j}$ is the flux of the reaction and ${S}_{i,j}$ is the stoichiometry matrix. The FVA was implemented in GAMS software^75^ and the resulting data was analyzed in R^79^. Furthermore, those reactions that had a minimum and maximum value of zero were filtered (blocked reactions) out and finally, the enzymes that catalyzed at least one non-blocked reaction were selected (non-zero).

Enzymes related to previously selected reactions were deleted one by one in the metabolic model and FBA was recalculated each time to determine their effect on the metabolic phenotype. Deletions were performed by setting upper and lower fluxes bounds to zero^80^. GEA was completed as previously described by Edwards and Palsson^80^.

Since genomes can present protein duplications and, therefore, enzymatic redundancy, deletions were also made in terms of clusters of enzymes based on sequence similarity. This clustering allows classifying each group into a specific enzyme. In this study, enzymatic groups were determined by CD-Hit^75^ with an identity threshold of 90%.

### Nomination of therapeutic targets

Three nomination criteria were defined in order to identify potential therapeutic targets: 1. Target enzymes must not have counterpart versions within the human proteome, which was expected to reduce the effect of inhibitors on host’s metabolism. This was verified by comparing the sequences of potential enzymes with those reported in the human genome using BlastP^81^, where the candidate enzymes were used as a query and the human proteome (UniProt: UP000005640) as the database. 2. Target enzymes were selected to be easily quantified (i.e. there are commercially available quantification kits), and 3. Inhibitors must be reported for selected enzymes in specialized databases such as BindingDB^82^ and BRENDA^83^.

### Heterologous expression of MpHCS, MpHSD, and MpSDH from *M. pachydermatis* in *E. coli*

Total RNA from *M. pachydermatis* CBS 1878 was isolated using TRIzol [Invitrogen] according to the manufacturer’s instructions^84^. Cells were homogenized with a TissueLyser [Qiagen]^85^ and total RNA was purified using RNeasy Mini Kit [Qiagen]^86^. 1 μg of purified RNA was reverse transcribed into cDNA using the SMARTer PCR cDNA Synthesis Kit [Clontech]^87^. The cDNA of HCS and HSD genes (*mphcs* and *mphsd*) were amplified by PCR Phusion^®^ DNA polymerase [NEB]^88^. Primers for cloning (Supplementary Table S4) were designed with 15 bp overlaps to the vector pET6xHN-N (Table 4). PCR products were purified using QIAquick PCR Purification Kit [Qiagen]^89^, then fused in-frame into the pre-linearized vector and transformed into *E. coli* strain BL21 (Table 4) for expression.

A synthetic MpSDH gene (ID MN527521, NCBI) flanked by the restriction enzymes *Nco*I and *Not*I was designed using the reported MpSDH gene from *M. pachydermatis* CBS 1879 as a template and synthetized by Shangai ShineGene Biotech, Inc.^90^. This synthetic gene was initially placed into the pUC57-*mpsdh* plasmid and later introduced into the final vector pET6xHN-C for expression in *E. coli* DH5α (Table 4)^90^. Plasmids were purified through GeneJET Plasmid Miniprep Kit [ThermoScientific]^91^, and digested with *Nco*I and *Not*I following standard protocols^92^. The *mpsdh* gene and the open pET6xHN-C were separated by agarose-electrophoresis and purified by GeneJET Gel Extraction Kit [Thermo Scientific]^93^ for subsequently ligation with NEB^94^ protocol and the plasmid pET6xHN-C-*mpsdh* ligation product was inserted into *E. coli* DH5α and into *E. coli* BL21 (Table 4) by heat shock transformation^95^.

Once *E. coli* strains BL21 were constructed (Table 4), growth and biomass profiles were established for each strain in order to determine the time in which an optimal optical density (OD) of 0.6–0.8 is reached and the approximate volume to obtain sufficient biomass to perform protein purification.

Expression was induced by the addition of 2 mM (final concentration) of isopropyl-d-1-thiogalactopyranoside (IPTG [Sigma; Calbiochem]) to cultures of 1200 mL in LB medium with an OD_600_ of 0.6–0.8. The cultures were maintained at 37 °C and 5 h after initiation of induction the biomass was recovered by centrifugation. The cells were re-suspended in lysis buffer (6.89 g/L NaH2PO4.H2O [Merck], 17.55 g/L NaCl [Merck], 100 µL/L Tween 20 [Sigma-Aldrich], 10 mL/L Triton X-100 [Panreac Quimica], pH 8.0) in a ratio 6 mL per 1 g of pellet and the lysis was carried out using a VibraCell^TM^ sonicator with an amplitude of 37% for 40 cycles of 40 seconds of dosing and 20 seconds of sonication. After, the protein purification was performed through immobilized metal affinity chromatography using Profinity^TM^ IMAC Resins [Bio-Rad]^96^ with Nickel charged resin (Nickel (II) chloride [Scharlau; Chemi]) following the instruction manual^96^. Finally, the protein concentration was quantified using a NanoDrop Thermoscientific^TM^ spectrophotometer and the proteins were separated by SDS-polyacrylamide gel electrophoresis (SDS-PAGE) on a 12.5% polyacrylamide gel under denaturing conditions and stained with Coomassie blue.

### Enzymatic activity

For the evaluation of the enzymatic activity of MpHSD and MpSDH, NAD/NADH quantification Test Kit [Sigma-Aldrich] protocol^24^ was used. The reverse reaction of MpHSD enzyme was evaluated by NADH detection using15 mM, 20 mM, 25 mM, 30 mM, and 35 mM ethanol as substrate and 5.22 mg/mL total protein concentration. For inhibition condition, the same concentrations of substrate plus 1 mM L-threonine [Sigma-Aldrich] was used. Both assays were detected using a StatFax 2100 [Wiener lab Group] following standard protocols^24^; MpSDH activity was also evaluated with the reverse reaction, employing 4.32/4.65 mg/mL total protein, 15 mM, 20 mM, 25 mM, 30 mM 2-OG substrate, in the presence or absence of 75 mM L-lysine [Sigma-Aldrich]. The NAD cycling buffer and NADH developer were not added in the master reaction mix but NADH standard was added. The colorimetric detection was made in Multiskan GO [Thermo Scientific] at 259 nm (detect NAD) and 340 nm (detect NADH) keeping the read time.

For MpHCS the Coenzyme A Test Kit [Sigma-Aldrich] protocol^25^ was used with 15 mM, 20 mM, 25 mM, 30 mM 2-OG [MERK] as substrate and 3.45 mg/mL enzyme. For the condition with inhibitor, 1 mM L-lysine [Sigma-Aldrich] was employed and the final volume was the same, adjusting the amount of Coenzyme A Assay Buffer. Finally, colorimetric detection was made using a Multiskan GO [Thermo Scientific] at 535 nm (λex), using horseradish peroxidase (3.23 mg/mL) to identify CoA product following standard protocols^25^.

### *In vitro* susceptibility tests

*In-vitro* susceptibility assays on broth microdilution and agar diffusion were performed to evaluate the inhibitory capacity of L-lysine and L-threonine on *M. pachydermatis* isolates (Table 3) and on the other reference strains, *M. furfur*, *M. sympodialis* and atypical *M. furfur* (Table 4). Broth microdilution methods were carried out according to the M27-A3 reference document of CLSI^97,98^. Inoculums were adjusted using Neubauer hemocytometer at 2 × 10^6^ CFU/mL, the medium used was modified Sabouraud broth^67^ and the concentrations employed (mg/mL) for L-lysine was from 1.5 to 4 and for L-threonine was from 16 to 25.

For the agar well diffusion method^99^, first a microbial suspension of 2 × 10^6^ CFU/mL was prepared for each strain and isolate followed by massive culture on a modified Dixon agar. Wells with a diameter of 8.5 mm was created in the medium and filled with 100 µL of the extract in concentrations (mg/mL) of 30, 40 and 50 of L-threonine or 50, 100, 150 and 200 of L-lysine and negative control with tween 80 (0.05%). Next, the plates were incubated at 33 °C for 3 days and results were recorded after 24, 48 and 72 hours. After that, the mean diameter of the inhibition zones was reported in millimeters and the inhibition was determined by comparing these to negative control (ultrapure water). The difference between the treatments was analyzed with ANOVA using the Tukey method^100^ (p-value < 0.05, confidence level 95%).

For microdilution method, the quality control included an AB test on *Candida krusei* and *Candida parapsilosis* strains (Table 4), as described in the CLSI M27A-A reference method. For the method of agar diffusion AB and Flz in solution or E-tests^®^ were used as a positive growth inhibition control.

### HEKa cell culture viability using MTT colorimetric assay

HEKa cells were grown to 80% confluence in Dulbecco’s Modified Eagle Medium (DMEM) in a cell culturing incubator with 5% CO_2_. Then, these cells were trypsinizated following the recommended procedure by ATCC^29^. After the trypsinization, the cell density was determined using a Neubauer hemocytometer with the purpose of seeding 10e4 cells in each well of a 96-well flat bottom plate in 100 µL of DMEM and plates were subsequently incubated for 1 day at 37 °C in 5% CO_2_. Then, 100 µL of 3 L-lysine solution with different concentration were added to a well, experiments were performed in triplicate. The 3 tested L-lysine concentrations were the same that caused the growth inhibition in the agar well diffusion method (200,150,100 mg/mL). The negative control consisted of 100 µL of DMEM plus 100 µL of cell culture, and the blank of DMEM alone. Then, the cells in the 96-well plate were incubated for 5 days under 5% CO_2_. At the 5th day, 10 uL of MTT was added to each well. At the 6th day, the absorbance was read at 562 nm using a flat bottom plate-reader. The absorbance was normalized to 100%, where 100% indicated that all cells were viable (normalized with the absorbance of the negative control minus the absorbance of the blank).

## Supplementary information

Supplementary information.

## Data Availability

All data generated or analyzed during this study are included in this published article (and its Supplementary Information Files).
