# Supplementary material for: New Therapeutic Candidates for the Treatment of Malassezia pachydermatis -Associated Infections
Source: Sci Rep. 2020 Mar 17;10:4860. doi: 10.1038/s41598-020-61729-1 (PMC7078309; doi:10.1038/s41598-020-61729-1)
Supplement: Supplementary file 1 — Supplementary information. [file 41598_2020_61729_MOESM1_ESM.pdf]

NEW THERAPEUTIC CANDIDATES FOR THE TREATMENT OF *Malassezia pachydermatis* -ASSOCIATED INFECTIONS

Angie Sastoque, Sergio Triana, Kevin Ehemann, Lina Suarez, Silvia Restrepo, Han Wösten, Hans de Cock, Miguel Fernández-Niño, Andrés González Barrios, Adriana Marcela Celis Ramírez

SUPPLEMENTARY INFORMATION

Supplementary Table S1: Biomass composition used as objective function in the metabolic network[28] with the metabolites with pathologies emphasized.

KEGG ID	Coefficient	Compound	METANETX ID	Pathology
C00001	-59,276	H2O	MNXM2	NO
C00002	-59,276	atp	MNXM3	NO
C00965	-11,348	1,3-beta-D-Glucan	MNXM6492	NO
C00096	-0.8079	GDP-mannose	MNXM82	NO
C00369	-0.5185	starch/glycogen	MNXM93732	NO
C00041	-0.4588	ala-L	MNXM32	NO
C00025	-0.3018	glu-L	MNXM89557	NO
C00049	-0.2975	asp-L	MNXM42	NO
C00123	-0.2964	leu-L	MNXM140	NO
C00037	-0.2904	gly	MNXM29	NO
C00047	-0.2862	lys-L	MNXM78	YES
C00183	-0.2646	val-L	MNXM199	YES
C00407	-0.1927	ile-L	MNXM231	YES
C00188	-0.1914	thr-L	MNXM142	YES
C00065	-0.1854	ser-L	MNXM53	YES
C00148	-0.1647	pro-L	MNXM114	YES
C00062	-0.1607	arg-L	MNXM70	YES
C00079	-0.1339	phe-L	MNXM97	YES
C00064	-0.1054	gln-L	MNXM37	YES
C00082	-0.102	tyr-L	MNXM76	NO
C00152	-0.1017	asn-L	MNXM147	NO
C00135	-0.0663	his-L	MNXM134	YES
C00105	-0.0599	ump	MNXM80	YES
C00073	-0.0507	met-L	MNXM61	YES
C00144	-0.046	amp	MNXM113	YES
C00020	-0.046	gmp	MNXM14	NO
C00055	-0.0447	cmp	MNXM31	NO

C00078	-0.0284	trp-L	MNXM94	YES
C01083	-0.0234	tre	MNXM198	YES
C00059	-0.02	SO4	MNXM58	NO
C00097	-0.0066	cys-L	MNXM55	YES
C00364	-0.0036	damp	MNXM257	YES
C00360	-0.0036	dtmp	MNXM432	NO
C00239	-0.0024	dcmp	MNXM266	NO
C00362	-0.0024	dgmp	MNXM546	NO
C05437	-0.0015	zymst	MNXM574	YES
C00255	-0.00099	rib	MNXM270	YES
C01694	-0.0007	ergst	MNXM922	NO
C00010	-0.000001	NAD	MNXM12	NO
C00575	-0.000001	CoA	MNXM243	NO
C00461	-0.000001	FAD	MNXM46301	NO
C00051	-0.000001	gthrd	MNXM57	YES
C00101	-0.000001	thf	MNXM79	NO
C00003	-0.000001	chitin	MNXM8	NO
C00016	-0.000001	camp	MNXM96415	NO
C00035	0.8079	GDP	MNXM30	NO
C00008	59,276	ADP	MNXM7	NO
C00009	59,305	Orthophosphate	MNXM9	NO
C00080	11,740,002	H	MNXM1	NO

Supplementary Table S2. Reactions added to the network after the manual curation.

ID	Compartment	Equation	Description	Source
MNXR3255	Cytoplasm	1 MNXM1036 + 4 MNXM2 + 1 MNXM39 + 3 MNXM5 = 2 MNXM1 + 3 MNXM4 + 1 MNXM482 + 3 MNXM6	1 `4,4-dimethyl-5alpha-cholesta-8,14,24-trien-3beta-ol` + 4 `H2O` + 1 `formate` + 3 `NADP(+)` = 2 `H(+)` + 3 `O2` + 1 `lanosterol` + 3 `NADPH`	rhea:25286
MNXR3256	Cytoplasm	1 MNXM130 = 1 MNXM482	1 `(S)-2,3-epoxysqualene` = 1 `lanosterol`	rhea:14621
MNXR56252	Cytoplasm	1 MNXM1804 + 1 MNXM5 = 1 MNXM1 + 1 MNXM36392 + 1 MNXM6	1 `4alpha-methyl-5alpha-cholesta-8,24-dien-3beta-ol` + 1 `NADP(+)` = 1 `H(+)` + 1 `4alpha-methyl-5alpha-cholesta-8,24-dien-3-one` + 1 `NADPH`	rhea:36379
MNXR65591	Cytoplasm	1 MNXM13 + 1 MNXM36392 + 1 MNXM6 = 1 MNXM37762 + 1 MNXM5	1 `CO2` + 1 `4alpha-methyl-5alpha-cholesta-8,24-dien-3-one` + 1 `NADPH` = 1 `4alpha-carboxyl-4beta-methyl-5alpha-cholesta-8,24-dien-3beta-ol` + 1 `NADP(+)`	rhea:33447
MNXR65590	Cytoplasm	2 MNXM1 + 3 MNXM4 + 3 MNXM6 + 1 MNXM913 = 4 MNXM2 + 1 MNXM37762 + 3 MNXM5	2 `H(+)` + 3 `O2` + 3 `NADPH` + 1 `4,4-dimethyl-5alpha-cholesta-8,24-dien-3beta-ol` = 4 `H2O` + 1 `4alpha-carboxyl-4beta-methyl-5alpha-cholesta-8,24-dien-3beta-ol` + 3 `NADP(+)`	rhea:33443
MNXR29289	Cytoplasm	1 MNXM1 + 1 MNXM10 + 1 MNXM2563 + 1 MNXM53 = 1 MNXM2 + 1 MNXM2623 + 1 MNXM29 + 1 MNXM8	1 `H(+)` + 1 `NADH` + 1 `tetrahydropteroyltri-L-glutamate` + 1 `L-serine` = 1 `H2O` + 1 `5-methyltetrahydropteroyltri-L-glutamate` + 1 `glycine` + 1 `NAD(+)`	seed:rxn12215
MNXR69079	Cytoplasm	1 MNXM1 + 1 MNXM1617 + 1 MNXM2 = 1 MNXM245	1 `H(+)` + 1 `1-pyrroline-5-carboxylate` + 1 `H2O` = 1 `L-glutamate 5-semialdehyde`	rhea:28234
MNXR31743	Cytoplasm	1 MNXM20 + 1 MNXM231 = 1 MNXM439 + 1 MNXM89557	1 `2-oxoglutarate` + 1 `L-isoleucine` = 1 `(S)-3-methyl-2-oxopentanoate` + 1 `L-glutamate`	rhea:24801
MNXR3145	Cytoplasm	1 MNXM1 + 1 MNXM426 + 1 MNXM6 = 1 MNXM5 + 1 MNXM734	1 `H(+)` + 1 `2-acetolactate` + 1 `NADPH` = 1 `NADP(+)` + 1 `2,3-dihydroxy-3-methylbutanoate`	kegg:R03051
MNXR32586	Cytoplasm	1 MNXM199 + 1 MNXM20 = 1 MNXM238 + 1 MNXM89557	1 `L-valine` + 1 `2-oxoglutarate` = 1 `3-methyl-2-oxobutanoate` + 1 `L-glutamate`	rhea:24813

MNXR56338	Cytoplasm	1 MNXM2 + 1 MNXM20 + 1 MNXM268 = 1 MNXM2 + 1 MNXM263 + 1 MNXM89557	1 `H2O` + 1 `2-oxoglutarate` + 1 `L-2-aminoadipate` = 1 `H2O` + 1 `2-oxoadipate` + 1 `L-glutamate`	biopath:RXN01183
MNXR13125	Cytoplasm	1 MNXM2378 + 1 MNXM77 = 1 MNXM417	1 `glyceraldehyde 3-phosphate` + 1 `glycerone phosphate` = 1 `D-fructose 1,6-bisphosphate`	rhea:14729
MNXR81572	Mithochondria	1 MNXM11 + 1 MNXM33 = 1 MNXM1 + 1 MNXM119 + 1 MNXM3	1 `diphosphate` + 1 `FAD` = 1 `H(+)` + 1 `FMN` + 1 `ATP`	rhea:17237
MNXR10784	Cytoplasm	1 MNXM26 + 1 MNXM320 = 1 MNXM2 + 1 MNXM497	1 `acetate` + 1 `phenol` = 1 `H2O` + 1 `phenylacetate`	rhea:17309
MNXR84842	Cytoplasm	1 MNXM1 + 1 MNXM2415 + 1 MNXM45 = 1 MNXM320 + 1 MNXM49	1 `H(+)` + 1 `phenyl sulfate` + 1 `adenosine 3',5'-bisphosphate` = 1 `phenol` + 1 `3'-phosphoadenylyl sulfate`	kegg:R01242
MNXR3900	Cytoplasm	1 MNXM1 + 1 MNXM11 + 1 MNXM432 = 1 MNXM2 + 1 MNXM286	1 `H(+)` + 1 `diphosphate` + 1 `dAMP` = 1 `H2O` + 1 `dATP`	rhea:28334
MNXR3902	Cytoplasm	1 MNXM1 + 1 MNXM11 + 1 MNXM257 = 1 MNXM2 + 1 MNXM394	1 `H(+)` + 1 `diphosphate` + 1 `dTMP` = 1 `H2O` + 1 `dTTP`	rhea:28534
MNXR3898	Cytoplasm	1 MNXM1 + 1 MNXM11 + 1 MNXM266 = 1 MNXM2 + 1 MNXM360	1 `H(+)` + 1 `diphosphate` + 1 `dCMP` = 1 `H2O` + 1 `dCTP`	rhea:22636
MNXR69877	Cytoplasm	1 MNXM1 + 1 MNXM6 + 1 MNXM89557 + 1 MNXM89905 = 1 MNXM2 + 1 MNXM384 + 1 MNXM5	1 `H(+)` + 1 `NADPH` + 1 `L-glutamate` + 1 `(S)-2-amino-6-oxohexanoate` = 1 `H2O` + 1 `L-saccharopine` + 1 `NADP(+)`	rhea:10020
MNXR7299	Cytoplasm	1 MNXM114 + 1 MNXM5 = 2 MNXM1 + 1 MNXM1617 + 1 MNXM6	1 `L-proline` + 1 `NADP(+)` = 2 `H(+)` + 1 `1-pyrroline-5-carboxylate` + 1 `NADPH`	rhea:14109
MNXR29715	Cytoplasm	1 MNXM10287 = 1 MNXM1397	1 1-(5-phospho-D-ribosyl)-5-[(5-phospho-D-ribosylamino)methylideneamino]imidazole-4-carboxamide(4-) = 1 5-[(5-phospho-1-deoxy-D-ribulos-1-ylamino)methylideneamino]-1-(5-phospho-D-ribosyl)imidazole-4-carboxamide	seed:rxn12992

Supplementary Table S3. Reactions which upper and lower bounds were modified after the manual curation.

ID	Compartment	Old bounds	New bounds
MNXR74425	Mitochondria	-1000;1000	0;1000
MNXR74425	Cytoplasm	-1000;1000	0;1000
MNXR74186	Cytoplasm	-1000;1000	0;1000
MNXR70061	Cytoplasm	-1000;1000	0;1000
MNXR69932	Cytoplasm	-1000;1000	0;1000
MNXR68605	Cytoplasm	-1000;1000	0;1000
MNXR68447	Cytoplasm	-1000;1000	0;1000
MNXR66179	Cytoplasm	-1000;1000	0;1000
MNXR53521	Cytoplasm	-1000;1000	0;1000
MNXR4195	Cytoplasm	-1000;1000	0;1000
MNXR39679	Mitochondria	-1000;1000	0;1000
MNXR39679	Cytoplasm	-1000;1000	0;1000
MNXR35885	Peroxisome	-1000;1000	0;1000
MNXR35885	Mitochondria	-1000;1000	0;1000
MNXR35885	Cytoplasm	-1000;1000	0;1000
MNXR2911	Cytoplasm	-1000;1000	0;1000
MNXR13313	Cytoplasm	-1000;1000	0;1000
MNXR13031	Cytoplasm	-1000;1000	0;1000

Supplementary Table S4. Primers sequences for cassettes designed in this study.

NAME	SEQUENCE	LENGTH (bp)
HCS-F	taaggcctctgtcgaATGGCGGGTGTAGGCG	31
HCS_R	cagaattcgcaagctTTACGCATCCCACATAGCCCG	36
HSD_F	taaggcctctgtcgaATGGAGTCTGTGGTACAGC	34
HSD_R	cagaattcgcaagctTTAGCCACGGCGCTCC	31

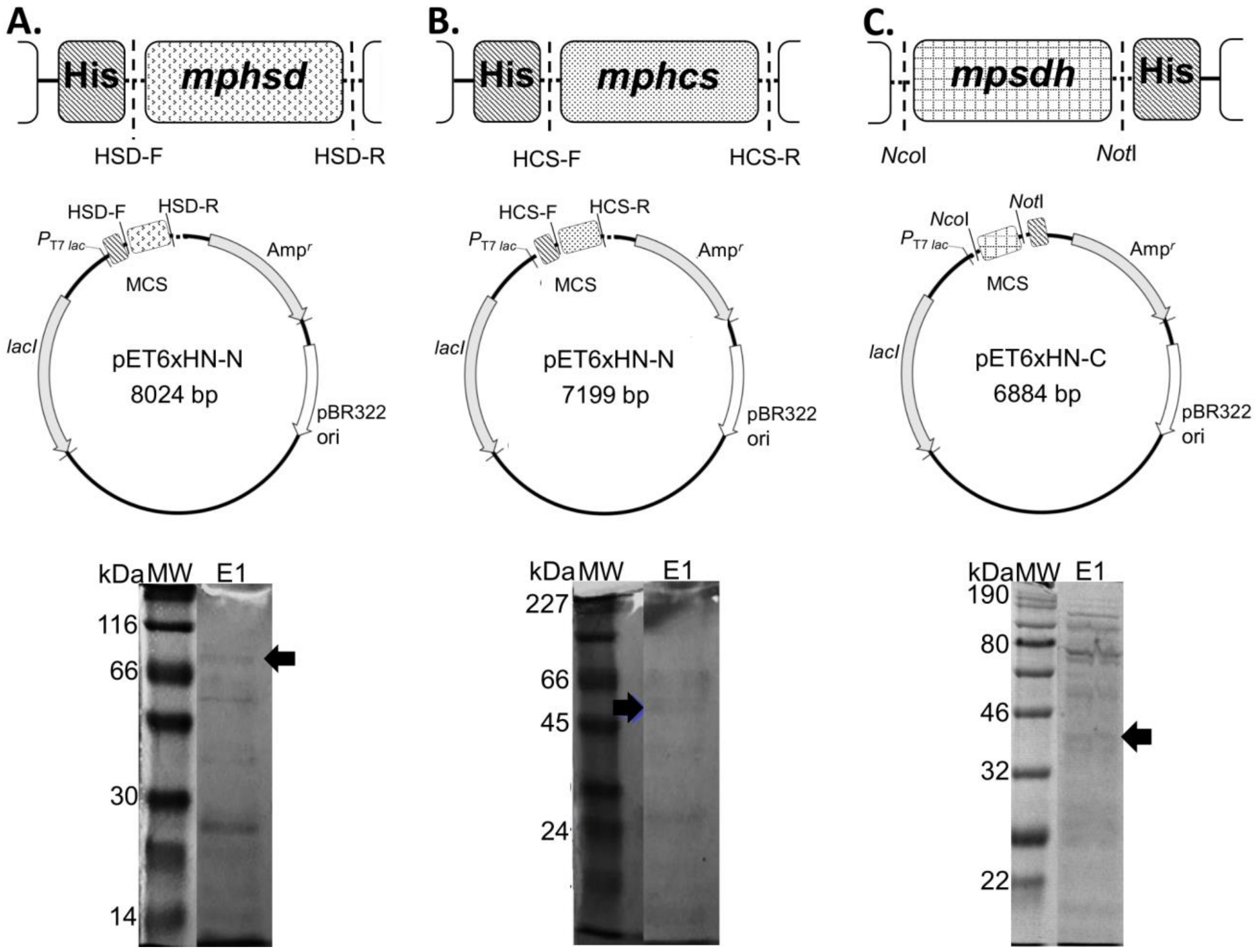

Supplementary Figure S1. Expression cassettes and cropped pictures of SDS-PAGE of MpHSD, MpHCS and MpSDH elutions. **A.** Topology of expression cassette and plasmid used for the heterologous expression of MpHSD as detected after HIS-tag purification and SDS PAGEas a band of 81 kDa (lane E1 black arrow). **B.** Topology of expression cassette and plasmid used for the heterologous expression of MpHCS as detected after HIS-tag purification and SDS PAGE as a band of 51 kDa (lane E1 black arrow). **C.** Topology of expression cassette and plasmid used for the heterologous expression of MpSDH as detected after HIS-tag purification and SDS PAGEas a band of 41 kDa (lane E1 black arrow). The expression cassettes with *mphsd* and *mphcs* CDS were flanked by the oligonucleotide sites, while *mpsdh* CDS was flanked by restriction enzymes. Cropped pictures from three different SDS-PAGE gels are shown. Full-length (uncropped) gels are presented in Supplementary Fig. S2.

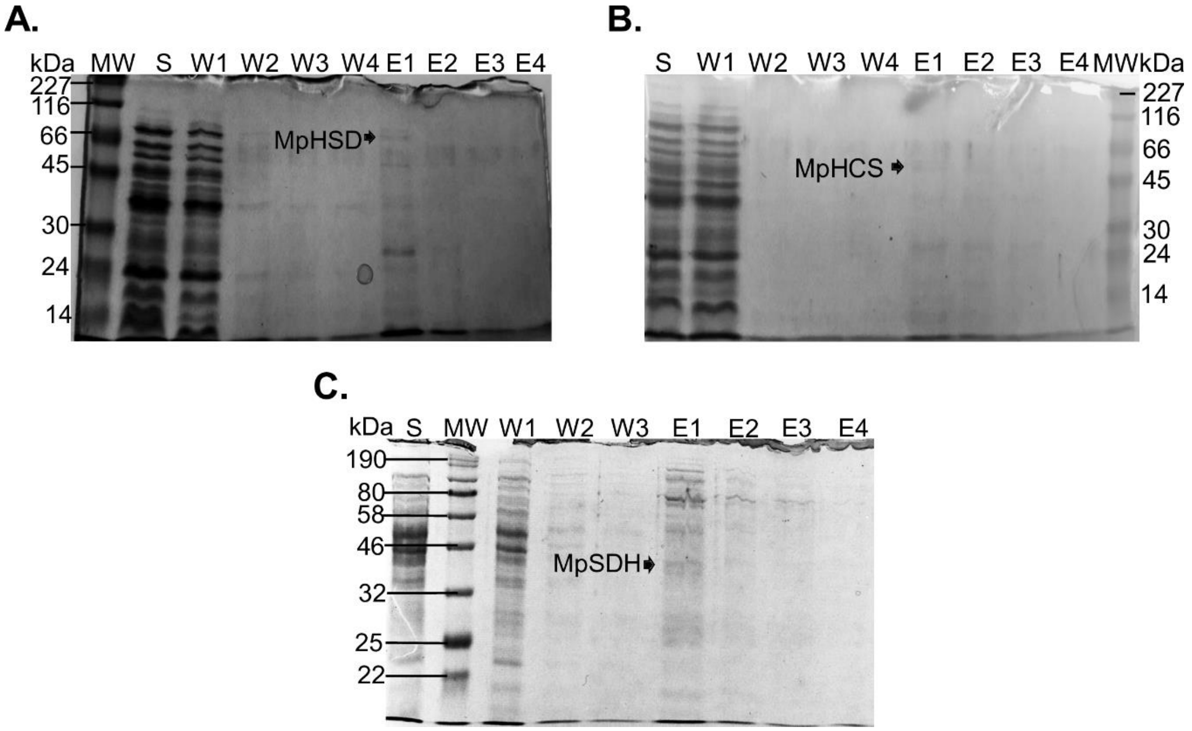

Supplementary Figure S2. Full-length SDS-PAGE gels for purified expressed proteins (**A**) from *E. coli* BL21 with pET6xHN-N-*mphsd*; (**B**) from *E. coli* BL21 with pET6xHN-N-*mphcs* and (**C**) from *E. coli* BL21 with pET6xHN-N-*mpsdh*. Enzymes of interest indicated with black arrows. MW, molecular weight; S, sample; W, washed; E, elution. Full-length (uncropped) gels are presented. Pictures were recorded using a Gel Doc™ EZ System (Bio-Rad Laboratories, USA).

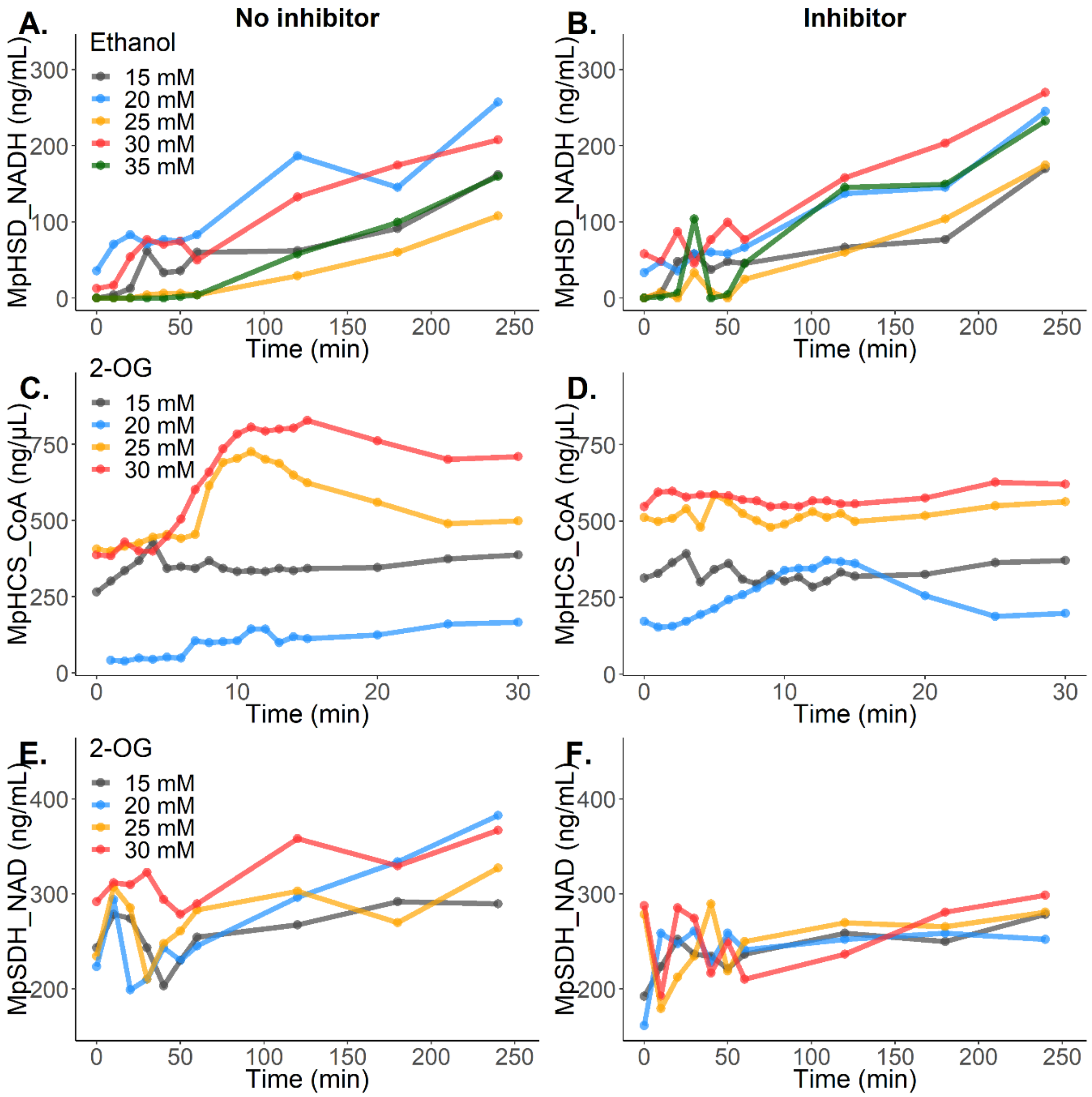

Supplementary Figure S3. Evaluation of the inhibitory capacity of amino acids upon candidates as therapeutic targets. **A.** Enzymatic activity of HSD with ethanol as substrate and NADH as reaction indicator. **B.** Enzymatic activity of HSD with ethanol as substrate, NADH as reaction indicator and L-threonine 1 mM as an inhibitor. **C.** Enzymatic activity of HCS with 2-OG as substrate and CoA as reaction indicator. **D.** Enzymatic activity of HCS with 2-OG as substrate, CoA as reaction indicator and L-lysine 1 mM as inhibitor. **E.** Enzymatic activity of SDH with 2-OG as substrate and NAD as reaction indicator. **F.** Enzymatic activity of SDH with 2-OG as substrate, NAD as reaction indicator and L-lysine 75 mM as inhibitor. Representative results of two biological replicates.

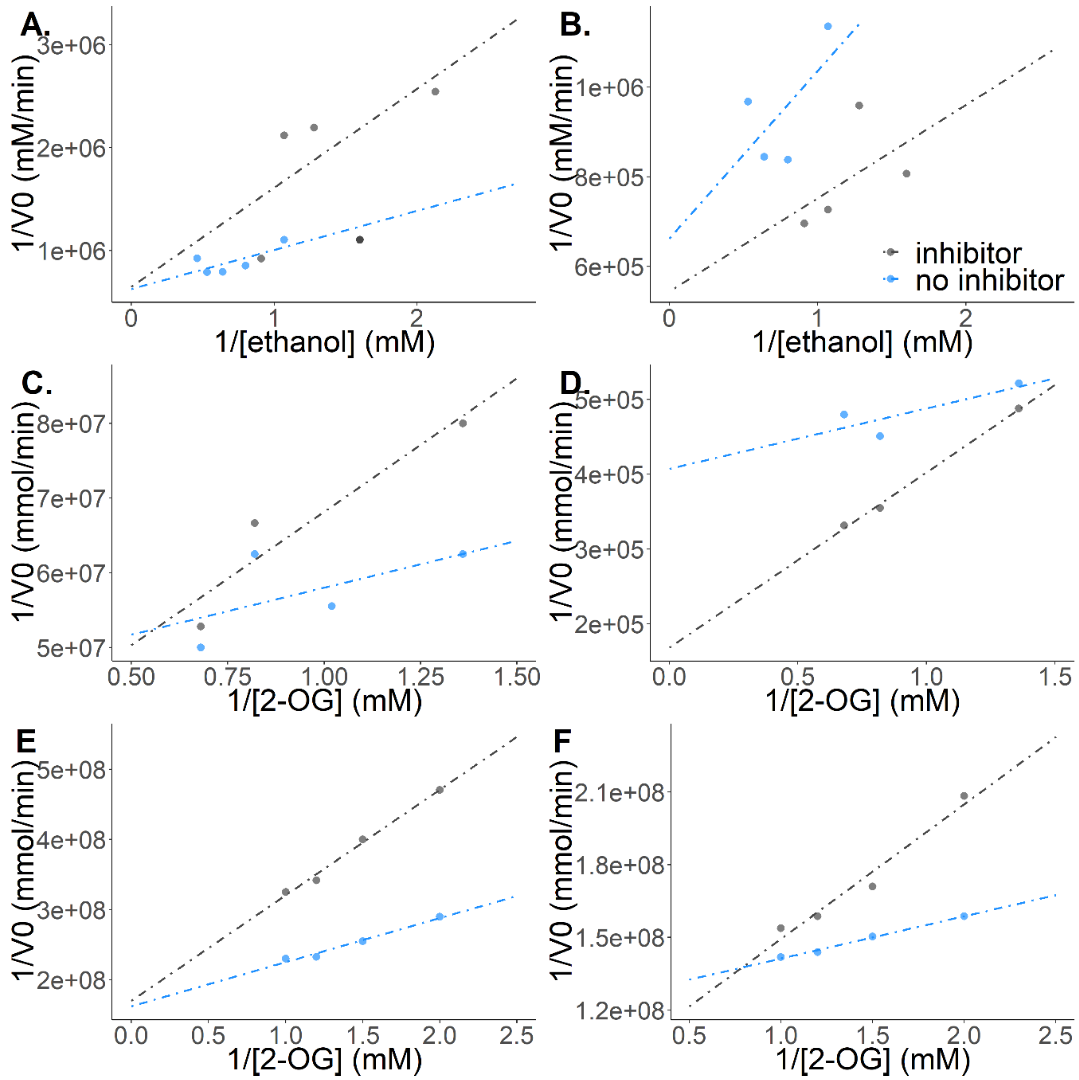

Supplementary Figure S4. Analysis of enzymatic activity assays by *Lineweaver-Burk* diagrams. **A., B.** Initial velocity pattern obtained for ethanol in different concentrations with NAD and MpHSD in a fixed concentration, compared to the inhibition of the substrate in the presence of 1 mM L-threonine. **C.,D.** Initial velocity pattern obtained for 2-OG in different concentrations with AcetylCoA and MpHCS at a fixed concentration, compared to substrate inhibition in the presence of 1 mM L-lysine. **E.,F.** Initial velocity pattern for 2-OG in different concentrations with NADH and MpSDH at a fixed concentration, compared to substrate inhibition in the presence of 75 mM L-lysine. The data shown are values for two biological replicates of each enzyme.

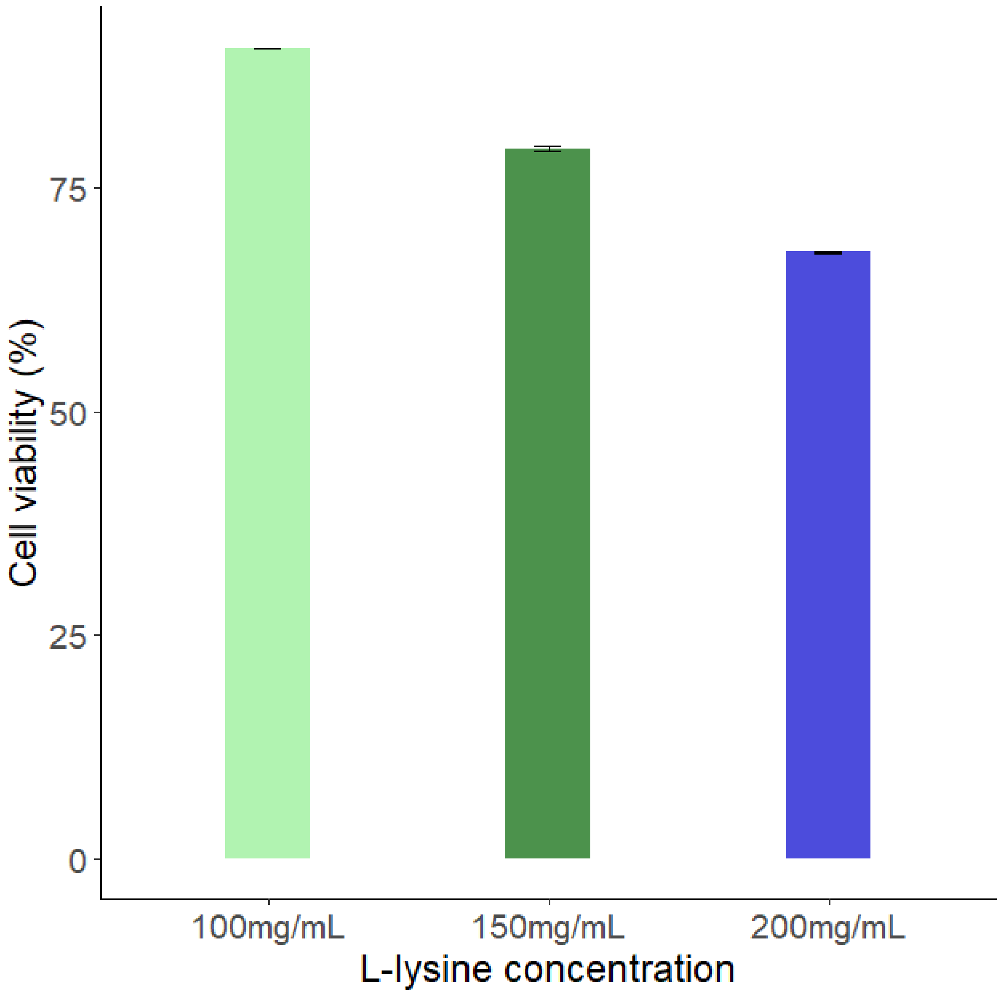

Supplementary Figure S5. MTT cell viability assay evaluating the cytotoxic activity of L-lysine on HEKa cells. The viability percentage with standard deviations is shown for each concentration. Experiments were performed 3 times.
